# Supplementary material for: Content-rich biological network constructed by mining PubMed abstracts
Source: BMC Bioinformatics. 2004 Oct 8;5:147. doi: 10.1186/1471-2105-5-147 (PMC528731; doi:10.1186/1471-2105-5-147)
Supplement: Additional File 2 — The original results of the above study (non-essential files are deleted to keep the file size under the limit set by BMC bioinformatics). [file 1471-2105-5-147-S2.bz2 › chilibotAdditionalFile2/dip05/419ID7890768E10288/html/INHBA_ACVR2.html]

 


 **INHBA** and **ACVR2** 
  
Found 4 abstracts in PubMed, retrieved 4.  
 

 What does Google say? 
 PDF only 
| .edu only 

---

**Interactive relationship** (e.g. stimulation, inhibition, etc)

**Non-interactive relationship** (e.g. studied together, co-existance, homology, etc.)

- mRNA levels of inhibin beta A  [ **INHBA** ]  and beta B, activin receptor  [ **ACVR2** ]  II and IIB ActRII  [ **ACVR2** ]  and ActRIIB, and follistatin were studied by Northern hybridization in the fetal rat gonads and mesonephroi.  Ref: 8156918 Endocrinology, 1994
